# Current status of MSK radiology training: an international survey by the European Society of Musculoskeletal Radiology (ESSR) Young Club

**DOI:** 10.1186/s13244-021-01070-x

**Published:** 2021-09-09

**Authors:** Domenico Albano, Danoob Dalili, Florian A. Huber, Ziga Snoj, Ana Vieira, Carmelo Messina, the Young Club of the European Society of Musculoskeletal Radiology (ESSR)

**Affiliations:** 1 IRCCS Istituto Ortopedico Galeazzi, Via Riccardo Galeazzi 4, 20161 Milan, Italy; 2 Sezione di Scienze Radiologiche, Dipartimento di Biomedicina, Neuroscienze e Diagnostica Avanzata, Università degli Studi di Palermo, Via Del Vespro 127, 90127 Palermo, Italy; 3 South West London Elective Orthopaedic Centre (SWLEOC), Dorking Road, Epsom, KT18 7EG UK; 4 School of Biomedical Engineering and Imaging Sciences, King’s College London, London, SE1 7EH UK; 5 Institute of Diagnostic and Interventional Radiology, University Hospital Zurich, Faculty of Medicine, University of Zurich, Raemistrasse 100, 8091 Zurich, Switzerland; 6 Institute of Radiology, University Medical Centre Ljubljana, Zaloška ul. 7, 1000 Ljubljana, Slovenia; 7 Porto Medical School – FMUP, São João University Hospital, Porto, Portugal

**Keywords:** Education, Radiology, Musculoskeletal diseases, Radiology, Interventional, Surveys and questionnaires

## Abstract

**Objectives:**

There is wide variation between Countries in the structures of residency programmes, need for subspecialisation, and health care system organisation. This survey was aimed at gathering information regarding current musculoskeletal (MSK) educational programmes offered both in European and non-European Countries.

**Methods:**

We administered an online survey to European Society of Radiology (ESR) residents and radiologists aged up to 35 years. The questionnaire was further disseminated by delegates of the ESR Radiology Trainees Forum. Survey consisted of 20 questions about the structure and organisation of MSK training programmes.

**Results:**

Overall, 972 participants from 86 Countries completed the survey, with a wide heterogeneity of answers. Of them, 636 were residents (65.9%), 329 were certified radiologists (34.1%), with a mean age of 30.8 ± 3 years. Almost half of the participants had a dedicated MSK rotation/block during residency, with a duration of 3–6 months in 62.5% of cases. A dedicated period in MSK Ultrasound was present in only one-third of residency programmes; 38% of participants were expected to learn interventional MSK procedures, but only 28.2% have been actively involved in interventions during their residency. Overall, 62.7% of participants rated the quality of their MSK training as poor to average. Almost all (93.1%) thought that MSK training could be improved in their residency, especially ultrasound practice (80.7%) and MRI reporting (71.1%).

**Conclusions:**

There are significant inconsistencies in the structure of MSK training offered by different Countries. Radiology trainees are showing substantial interest in MSK training, which necessitates strategic investments to standardise and enhance its quality.

## Key points

- MSK training was variable in content/structure both in European and non-European Countries
- Only half of responders underwent a dedicated MSK training during their residency programme
- About one-third of trainees had a dedicated period in MSK ultrasound

## Introduction

Over the past years, subspecialisation in radiology has become more important, owing to advancements in technology and increasing knowledge in all radiological fields [1]. This is particularly relevant within the subspecialty of musculoskeletal (MSK) radiology, as the expectations and demands of referring physicians and patients are ever-increasing [2, 3]. Moreover, an increasing number of patients are referred to radiology departments by highly subspecialised clinicians, who have developed their own practices to encompass gaining imaging expertise. Referring physicians can therefore form an independent opinion on imaging reports, whilst profiting from first-hand information deducted from the patient history and clinical examination. A recent study evaluated the role of subspecialised MSK radiologists in performing second-opinion consultations for MSK examinations, with up to 40% more discrepancies encountered in generalists’ reports [3]. In this scenario, high-quality education during residency is mandatory, both for improving patient care and the quality of radiological research [2, 4].

To meet such need to define and enhance the quality of MSK radiology education across Europe, the European Society of Radiology (ESR) published its Training Curriculum for Radiology, providing recommendations of all requirements suggested to calibrate and optimise MSK sub-specialist training [5]. Furthermore, the European Society of Musculoskeletal Radiology (ESSR) created the European Diploma in Musculoskeletal Radiology (EDiMSK), a qualification aimed at standardising, evaluating, and validating MSK training and expertise across Europe [6].

A wide variation between Countries exists in the structures of residency programmes, need for subspecialisation, and regarding the underlying health care system frameworks which govern the delivery of such training requirements. Nevertheless, there is no published data regarding the different models in which MSK training is delivered, how local training/teaching settings are organized, or what the expectations of residents are.

The ESSR Young Club was formed in 2017 during the annual ESSR meeting in Bari (Italy) with the purpose of putting together young radiologists, motivating them and creating a network of the future generation within the ESSR. To gain knowledge about the current situation of MSK training during residency, the ESSR Young Club promoted an international survey addressed at both European and non-European Countries. This paper reports the results of this survey, which aim is to provide a general overview of the organisation of MSK radiology training.

## Materials and methods

### Study design

The Education Committee of the ESR and the Executive Committee of the ESSR approved the distribution of this survey in October 2019. Consequently, a questionnaire was developed as a collaborative initiative by representatives of the ESSR Young Club. In line with previous studies, we used the free online tool “Google Forms” (Google LLC, Mountain View, CA, USA) to create and disseminate the survey, and collect answers [7, 8].

The anonymous survey was composed of 20 questions; the full list of questions and answers are displayed in Table 1. Eighteen questions required answers as unique or multiple-choice selections, whilst two questions requested entering a free-text response. Questions were addressed at assessing variations in clinical practice, available learning materials and methods, techniques and breadth of interventional training, as well as MSK-specific opportunities or challenges.

**Table 1:** Full list of questions included in the survey and pertinent answers from 972 participants

Question	Total answers	Answer
1. How old are you? (years)	968/972 (99.6%)	mean: 30.8 ± 3
		(range 23–35)
2. In which country did you receive (or you do receive) your radiology training?	971/972 (99.9%)	See Table 2
3. What is your country of origin?	971/972 (99.9%)	See Table 2
4. Are you a resident or certified radiologist?	965/972 (99.3%)	Residents: 636/965 (65.9%)
		Certified radiologists: 329/965 (34.1%)
5. What type of hospital do you work in?	965/972 (99.3%)	University hospital: 633/965 (65.7%)
		Larger community hospital: 179/965 (18.5%)
		Private practice: 87/965 (9%)
		Small community hospital: 66/965 (6.8%)
6. How is MSK training/teaching organized during your residency programme?	963/972 (99.1%)	We have a dedicated rotation/block for radiological subspecialties, including MSK: 462/963 (48%)
		We learn MSK radiology scattered over the various years of residency: 286/963 (29.7%)
		No specialist MSK training is available in our programme: 215/963 (22.3%)
7. If a dedicated training period in MSK exists, what is the duration?	459/462 (99.3%)	Between 3 and 6 months: 287/459 (62.5%)
		Less than 3 months: 112/459 (24.4%)
		Between 6 months and 1 year: 43/459 (9.4%)
		More than 1 year: 17/459 (3.7%)
8. How is it organized?	737/972 (75.8%)	Trainees rotate between the different MSK modalities: 523/737 (71%)
		Trainees are assigned to a specific modality (MRI, Ultrasound, etc.): 214/737 (29%)
9. Do you have/have you had a dedicated period in MSK Ultrasound during your residency?	967/972 (99.5%)	No: 647/967 (66.9%)
		Yes: 320/967 (33.1%)
10. How many hours of MSK related teaching do you receive annually in your residency programme?	954/972 (98.1%)	< 20 h: 632/954 (66.2%)
		20–40 h: 201/954 (21.1%)
		> 40 h: 121/954 (12.7%)
11. Who plays/played a role in your daily MSK training?	945/972 (97.2%)	Senior consultants: 558/945 (59%)
		Fellows (Junior specialist): 160/945 (16.9%)
		Other residents: 149/945 (15.8%)
		University Professor: 78/945 (8.2%)
12. How would you rate your overall MSK training?	969/972 (99.7%)	Poor: 316/969 (32.6%)
		Average: 282/969 (29.1%)
		Good: 173/969 (17.8%)
		Very good: 155/969 (16%)
		Excellent: 43/969 (4.4%)
13. How would you prefer to learn MSK radiology? (multiple choice)	970/972 (99.8%)	Daily practice on the job (with supervision): 870/970 (89.7%)
		Educational courses/Meetings: 496/970 (51.1%)
		Clinical case based presentation: 432/970 (44.5%)
		E-learning platforms: 426/970 (43.9%)
14. During your training period are/were you expected to learn interventional MSK procedures?	964/972 (99.2%)	No: 598/964 (62%)
		Yes: 366/964 (38%)
15. How do/did you participate in interventional MSK procedures during your residency?	950/972 (97.7%)	No MSK procedures are performed in my Institution: 351/950 (36.9%)
		As Observer: 331/950 (34.8%)
		Actively involved: 268/950 (28.2%)
16. Do you think that MSK training should/could be improved in your residency programme?	964/972 (99.2%)	Yes: 898/964 (93.1%)
		No: 66/964 (6.9%)
17. If yes, which part of MSK training should be improved? (multiple choice)	890/898 (99.1%)	Practice in ultrasound: 718/890 (80.7%)
		MR interpretation and case reporting in daily practice: 633/890 (71.1%)
		MSK case presentations and discussion: 543/890 (61%)
		Practice in ultrasound guided procedures: 523/890 (58.8%)
		Practice in CT guided procedures: 455/890 (51.1%)
		Educational courses/Meetings: 448/890 (50.3%)
		CT interpretation and case reporting: 446/890 (50.1%)
		Formal lecture based teaching lessons: 410/890 (45.2%)
		E-learning platforms: 395/890 (44.4%)
		Practice in fluoroscopy guided procedures: 332/890 (37.3%)
18. Are you informed about the ESR European Training Curriculum for Radiology?	964/972 (99.2%)	Not aware: 411/964 (42.6%)
		Aware but I have never read it: 411/964 (42.6%)
		I am well informed: 142/964 (14.8%)
19. Are you aware of the ESSR diploma in MSK radiology?	970/972 (99.8%)	Not aware but interested: 461/970 (47.5%)
		Aware and interested in sitting the exam in the future: 242/970 (24.9%)
		Aware and not interested: 130/970 (13.4%)
		Not aware and not interested: 123/970 (12.7%)
		Aware and I am working towards sitting the exam: 11/970 (1.1%)
		I have the Diploma: 3/970 (0.3%)
20. Do you think that this certification will be taken into consideration when applying for a job?	970/972 (99.8%)	Yes: 474/970 (48.9%)
		Not sure: 364/970 (37.5%)
		No: 132/970 (13.6%)

On February 4, 2020, an invitation to participate in the survey was distributed via email to all ESR members up to 35 years of age. This threshold was chosen as 35 years is the current upper age limit to be eligible for the ESSR Young Club. In addition, a link to the survey was made available on the ESR home web page. The survey was further supported by the Radiology Trainees Forum (RTF)—an ESR subcommittee dedicated to supporting and representing the views of radiology residents across Europe [9]. After two weeks, an email was sent to all RTF national delegates, as a reminder to flag up the survey to their respective Countries. The initially proposed period of open survey was four weeks. Hence, preliminary data of this survey were being expected to be presented during the 2020 European Congress of Radiology (ECR) in Vienna, Austria. However, due to the global COVID-19 pandemic [10] a subsequent postponement of the congress was announced during our survey period, as international lockdown measures in the majority of ECR participating Countries affected many speakers and radiology representatives. Following unanimous decision, the authors decided to conclude the survey after eight weeks.

Institutional Review Board approval was not required for this survey, as it did not involve patient related data.

### Data analysis

Data was collected and tabulated via Google Forms. Results were analysed by two radiologists with previous expertise in survey studies (C.M., D.A.). In general, descriptive statistics were used. Data and response rates were expressed as means and percentages. We further analysed data according to the different hospital settings (university hospital, larger community hospital, private practice and small community hospital) and to the top five Countries with the highest response rates.

## Results

We received a total of 973 replies. Manual review of the database resulted in exclusion of one survey reply, as no data were submitted. Thus, a total of 972 records from 86 Countries were included in our analysis. The absolute number of completed answers, stratified by country of origin and residency, are reported in Table 2.

**Table 2:** The absolute number of completed answers stratified by country of origin and residency

Country	N^o^ of answers (Country of residency)	N^o^ of answers (Country of origin)
Italy	172	171
UK	108	87
India	95	98
Spain	70	56
Portugal	58	60
Croatia	36	38
Slovenia	35	31
Turkey	35	32
Germany	27	34
Netherlands	22	23
Poland	22	22
Switzerland	20	8
Romania	19	23
Russia	17	17
Estonia	16	16
Austria	15	13
Bulgaria	15	15
Brazil	12	12
Sweden	12	6
Lithuania	10	10
Mexico	10	10
Pakistan	10	17
Colombia	7	13
Latvia	7	10
Tanzania	7	7
Egypt	6	7
Peru	6	7
Philippines	6	6
Ukraine	6	6
Greece	5	6
Hungary	5	5
Serbia	5	7
Argentina	4	3
France	4	3
Mongolia	4	4
Belarus	3	3
Iran	3	3
Ireland	3	7
Lebanon	3	4
Tunisia	3	4
USA	3	1
Algeria	2	2
Denmark	2	2
Ghana	2	2
Iraq	2	4
Kazakhstan	2	2
Kenya	2	2
Morocco	2	2
Myanmar	2	2
Norway	2	1
Panama	2	2
Armenia	1	1
Australia	1	0
Belgium	1	0
Cambodia	1	1
Cameroon	1	1
Canada	1	2
Chile	1	3
Czech Republic	1	2
Dominican Republic	1	1
Ecuador	1	1
Finland	1	1
Hong Kong	1	0
Jamaica	1	1
Kuwait	1	1
Libya	1	0
Nepal	1	1
Nicaragua	1	1
Nigeria	1	1
Palestine	1	1
Saudi Arabia	1	1
Singapore	1	2
Slovakia	1	2
South Korea	1	1
Sudan	1	1
Thailand	1	1
Uzbekistan	1	1
Afghanistan	0	1
Cyprus	0	1
Venezuela	0	2
Taiwan	0	1
Rep. of Moldova	0	1
Puerto Rico	0	1
Malaysia	0	7
Guatemala	0	2
Azerbaijan	0	2
Total	972	972

In total, 636/965 (65.9%) of answers were received by residents and 329/965 (34.1%) from board-certified radiologists, with an overall mean age of 30.8 ± 3 years (range: 23–35). The mean age of residents was 29.7 ± 2.5 years, while board-certified radiologists had a mean age of 32.2 ± 2.1. Regarding the hospital settings for board-certified radiologists, 140/329 (42.5%) of them reported to work at university hospitals, 75/329 (22.8%) at larger community hospitals, 76/329 (32.1%) at private practice, and 38/329 (11.6%) at small community hospitals. For residents, 493/636 (77.5%) were training at university hospitals, 104/636 (16.3%) at larger community hospitals, 11/636 (1.7%) in private practices, and 28/636 (4.4%) at small community hospitals.

Almost half of the participants (*n* = 462/963, 48%) had dedicated rotations for radiological subspecialties, including MSK, with the majority (*n* = 287/459, 62.5%) reporting a duration between 3 and 6 months. During MSK training, *n* = 523/737 residents reported rotation through the different MSK modalities (71% of cases), while a dedicated period in MSK ultrasound was scheduled in only 320/967 (33.1%) of residency programmes. A large number of participants (*n* = 632/954, 66.2%) reported that during their residency less than 20 h per year were dedicated to MSK-related teaching. Consultants played a major role in MSK training (*n* = 558/945, 59% of cases), while university professors in *n* = 78/945 (8.2%). In the remaining cases, the training was done by fellows (*n* = 160/945, 16.9) and other residents (*n* = 149/945, 15.8%). Overall, 598/969 (61.7%) of the participants rated the quality of their overall MSK training as poor to average (rated on a five-point Likert scale from poor to excellent).

Concerning interventional procedures, 366/964 (38%) of participants were expected to learn interventional MSK procedures, whilst only 268/950 (28.2%) have been actively involved in interventions during their residency.

Regarding the preferred teaching modality, the majority of participants (*n* = 870/970, 89.7%) declared that they would prefer to learn MSK radiology through daily practice, on the job training under supervision. Almost all survey participants (*n* = 898/964, 93.1%) wished for an overall improved MSK training during their residency programme, with a focus on ultrasound (*n* = 718/890, 80.7%), MRI reporting (*n* = 633/890, 71.1%), MRI case-based discussion (*n* = 543/890, 61%), and practice in ultrasound-guided (*n* = 523/890, 58.8%) and CT-guided procedures (455/890, 51.1%), respectively.

**Fig. 1:**
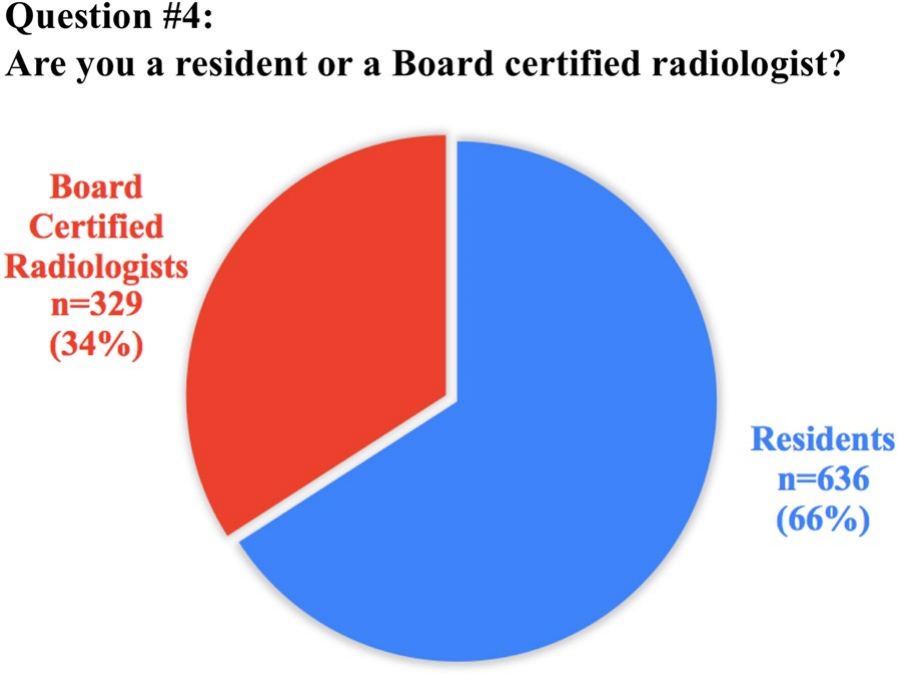
Graphical representation of answer distribution to question #4: percentage of residents versus board-certified radiologists

**Fig. 2:**
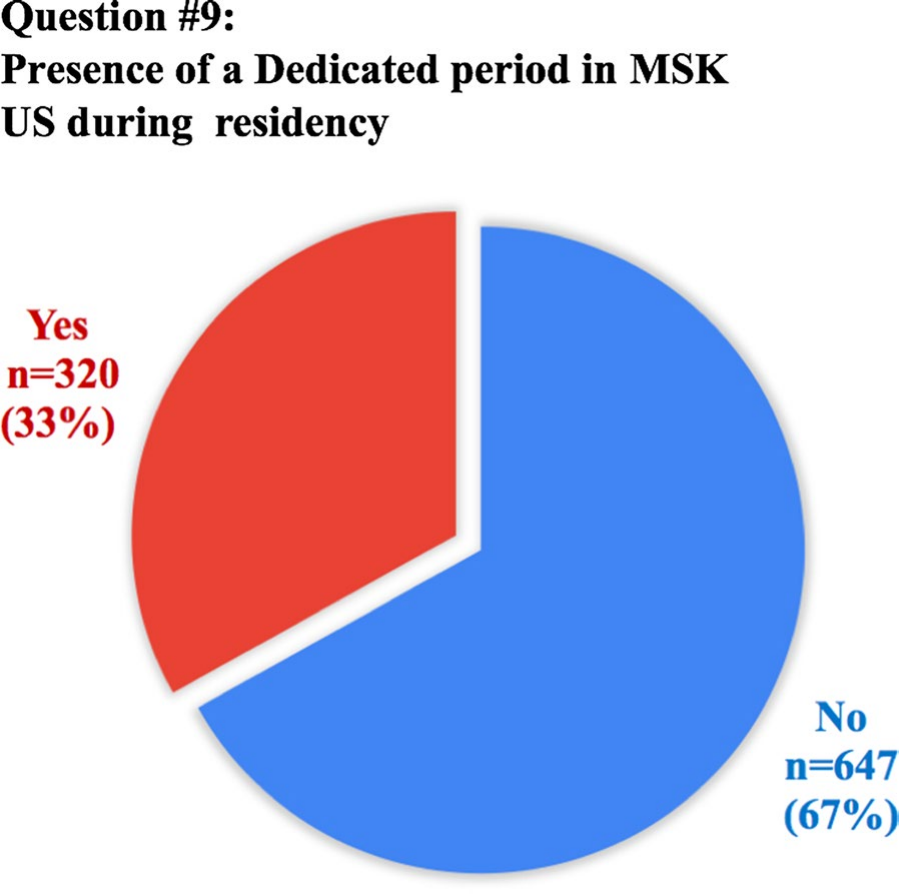
Graphical representation of answer distribution to question #9: presence of a dedicated period of MSK US

**Fig. 3:**
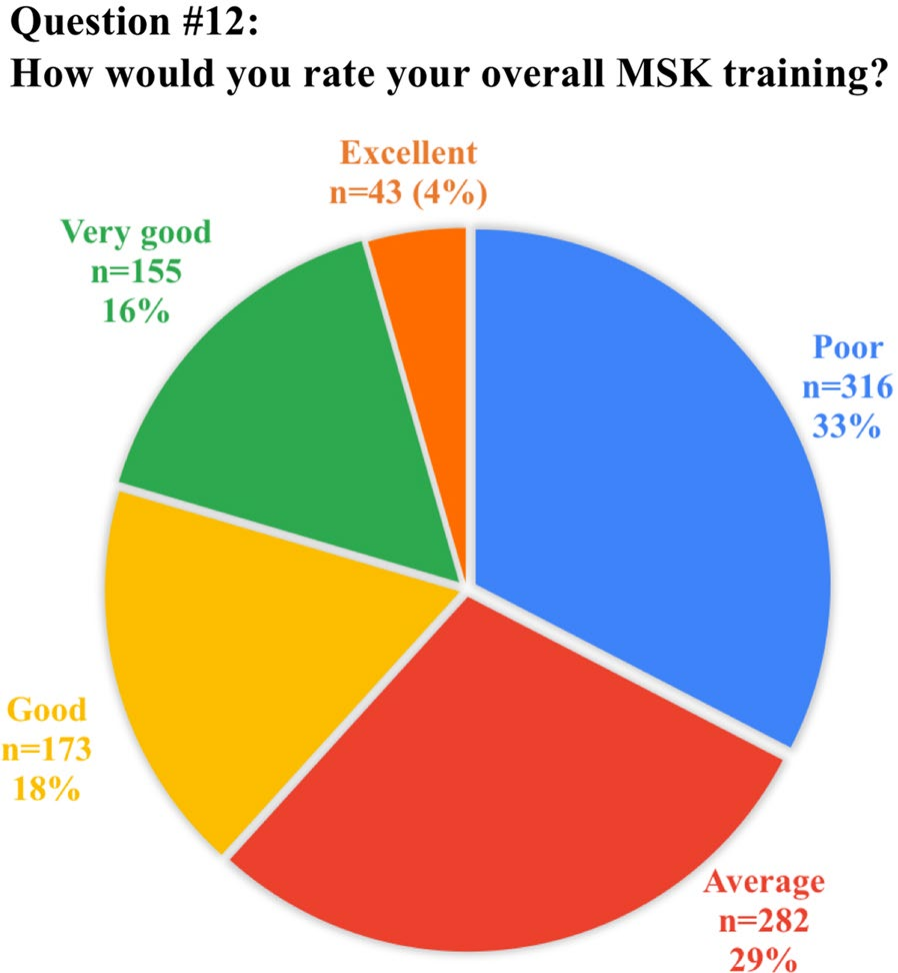
Graphical representation of answer distribution to question #12: overall rating of MSK training quality

**Fig. 4:**
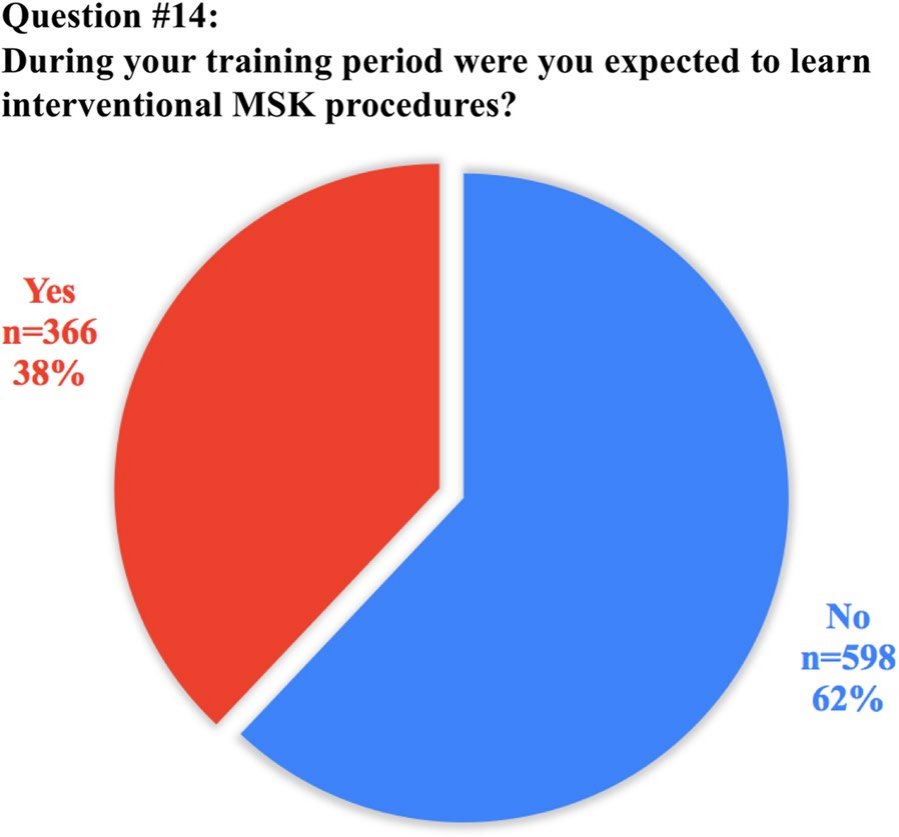
Graphical representation of answer distribution to question #14: possibility of learning MSK interventional procedures

**Fig. 5:**
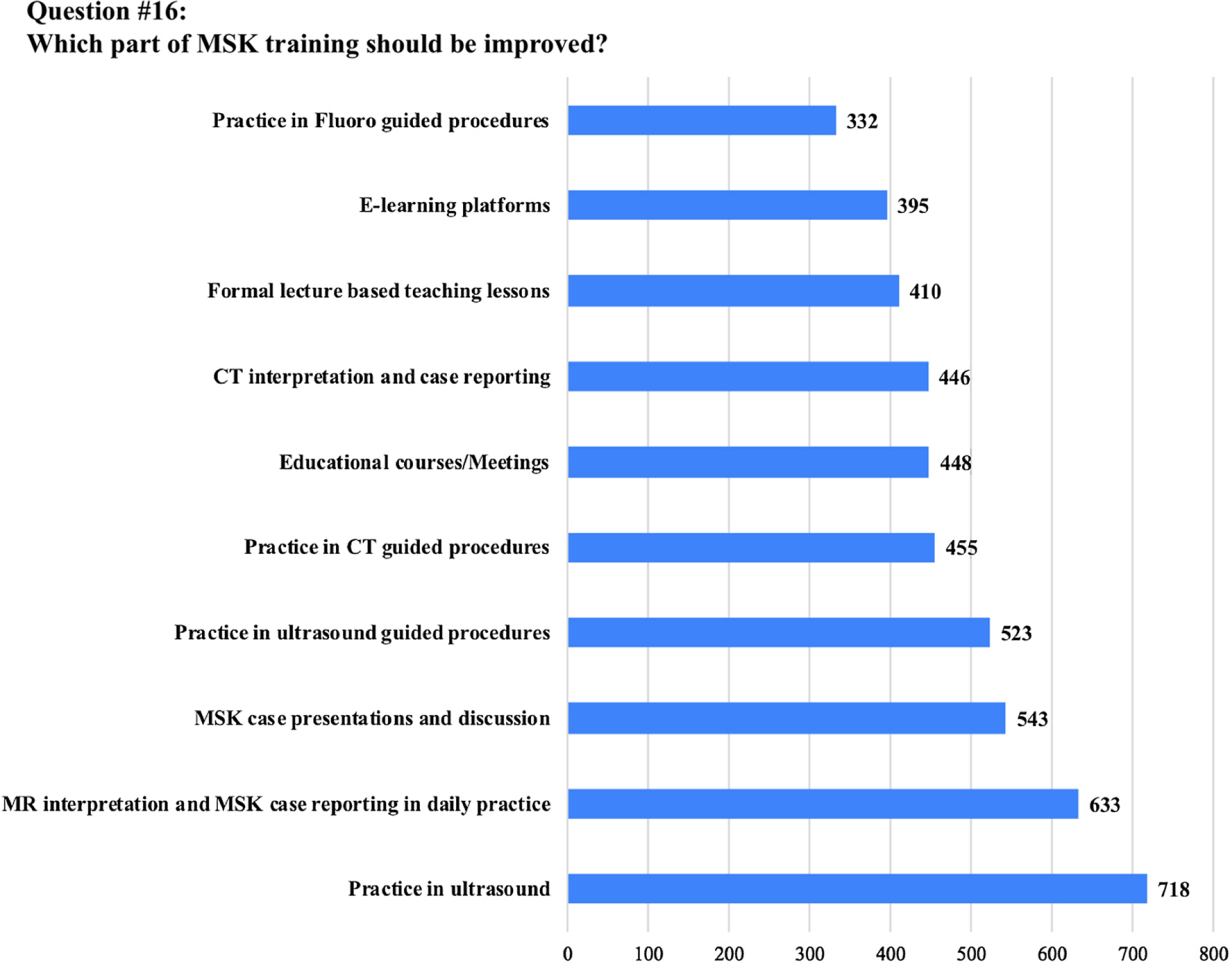
Graphical representation of answer distribution to question #16: need for improvement of MSK training period

When asked about the ESR European Training Curriculum for Radiology and of the EDiMSK, *n* = 411/964 (42.6%) and *n* = 461/970 (47.5%) of participants, respectively, declared that they were not aware of their existence; nevertheless, *n* = 474/970 (48.9%) of those surveyed thought that the EDiMSK could be taken into consideration when applying for a job. A summary of all results per question is presented in Table 1. Also, questions #4 (percentage of residents versus board-certified radiologists), #9 (presence of a dedicated period of MSK US), #12 (overall rating of MSK training quality), #14 (possibility of learning MSK interventional procedures), and #16 (need for improvement of MSK training period) are graphically represented in Figs. 1, 2, 3, 4 and 5, respectively.

The top five represented Countries of residency were Italy (*n* = 172/971, 17.7%), UK (*n* = 108/971, 11.1%), India (*n* = 95/971, 9.8%), Spain (*n* = 70/971, 7.2%), and Portugal (*n* = 58/971, 6%). The stratified responses from the aforementioned five Countries to the questions that yielded the most heterogeneity of results are reported in Table 3.

**Table 3:** Table 3 shows the 7 questions with the most heterogeneity of answers, according to the top five Countries represented in this survey

Question	Italy	UK	India	Spain	Portugal
6. How is MSK training/teaching organized during your residency programme?	We have a dedicated rotation/block for radiological subspecialties, including MSK: 73/171 (42.7%)	We have a dedicated rotation/block for radiological subspecialties, including MSK: 87/107 (81.3%)	No specialist MSK training is available in our programme: 46/92 (50%)	We have a dedicated rotation/block for radiological subspecialties, including MSK: 70/70 (100%)	We have a dedicated rotation/block for radiological subspecialties, including MSK: 24/58 (41.4%)
	We learn MSK radiology scattered over the various years of residency: 51/171 (29.8%)	We learn MSK radiology scattered over the various years of residency: 18/107 (16.8%)	We learn MSK radiology scattered over the various years of residency: 40/92 (43.5%)	We learn MSK radiology scattered over the various years of residency: 0/70 (0%)	We learn MSK radiology scattered over the various years of residency: 22/58 (37.9%)
	No specialist MSK training is available in our programme: 47/171 (27.5%)	No specialist MSK training is available in our programme: 2/107 (1.9%)	We have a dedicated rotation/block for radiological subspecialties, including MSK: 6/92 (6.5%)	No specialist MSK training is available in our programme: 0/70 (0%)	No specialist MSK training is available in our programme: 12/58 (20.7%)
9. Do you have/have you had a dedicated period in MSK Ultrasound during your residency?	No: 115/172 (66.9%)	Yes: 64/107 (59.8%)	No: 88/92 (95.7%)	Yes: 58/70 (82.9%)	No: 42/58 (72.4%)
	Yes: 57/172 (33.1%)	No: 43/107 (40.2%)	Yes: 4/92 (4.3%)	No: 12/70 (17.1%)	Yes: 16/58 (27.6%)
10. How many hours of MSK related teaching do you receive annually in your residency programme?	< 20 h: 138/168 (82.1%)	< 20 h: 74/107 (69.2%)	< 20 h: 60/90 (66.7%)	< 20 h: 32/70 (45.7%)	< 20 h: 46/58 (79.3%)
	20–40 h: 21/168 (13.5%)	20–40 h: 25/107 (23.4%)	20–40 h:52/90 (57.8%)	20–40 h: 26/70 (37.1%)	> 40 h: 10/58 (17.2%)
	> 40 h: 9/168 (5.4%)	> 40 h: 8/107 (7.5%)	> 40 h: 8/90 (8.9%)	> 40 h: 12/70 (17.1%)	20–40 h: 2/58 (3.4%)
12. How would you rate your overall MSK training?	Poor: 92/172 (53.5%)	Average: 36/107 (33.6%)	Average: 52/94 (57.8%)	Average: 20/70 (28.6%)	Poor: 30/58 (51.7%)
	Average: 35/172 (20.3%)	Very good: 34/107 (31.8%)	Poor: 36/94 (38.3%)	Very good: 16/70 (22.8%)	Average: 10/58 (17.2%)
	Good: 21/172 (12.2%)	Good: 15/107 (14%)	Good: 16/94 (9.7%)	Good: 20/70 (28.6%)	Good: 8/58 (13.8%)
	Very good: 17/172 (9.9%)	Poor: 14/107 (13.1%)	Very good: 0/94 (0%)	Excellent: 8/70 (11.4%)	Very good: 8/58 (13.8%)
	Excellent: 7/172 (4.1%)	Excellent: 8/107 (7.5%)	Excellent: 0/94 (0%)	Poor: 6/70 (8.6%	Excellent: 2/58 (3.4%)
14. During your training period are/were you expected to learn interventional MSK procedures?	No: 110/171 (64.3%)	No: 53/105 (50.5%)	No: 76/92 (82.6%)	Yes: 44/68 (64.7%)	No: 44/58 (75.9%)
	Yes: 61/171 (35.7%)	Yes: 52/105 (49.5%)	Yes: 16/92 (17.4%)	No: 24/68 (35.3%)	Yes: 14/58 (24.1%)
15. How do/did you participate in interventional MSK procedures during your residency?	No MSK procedures are performed in my Institution: 75/167 (44.9%)	Actively involved: 65/101 (64.3%)	No MSK procedures are performed in my Institution: 54/92 (58.7%)	Actively involved: 32/70 (45.7%)	No MSK procedures are performed in my Institution: 26/58 (44.8%)
	As Observer: 66/167 (39.5%)	As Observer: 36/101 (35.7%)	As Observer: 28/92 (8.7%)	As Observer: 30/70 (42.9%)	As Observer: 24/58 (41.4%)
	Actively involved: 26/167 (15.6%)	No MSK procedures are performed in my Institution: 0/101 (0%)	Actively involved: 10/92 (10.9%)	No MSK procedures are performed in my Institution: 8/70 (11.4%)	Actively involved: 8/58 (13.8%)
16. Do you think that MSK training should/could be improved in your residency programme?	Yes: 162/171 (94.7%)	Yes: 87/105 (82.9%)	Yes: 92/92 (100%)	Yes: 72/70 (88.6%)	Yes: 58/58 (100%)
	No: 9/171 (5.3%)	No: 18/105 (17.1%)	No: 0/92 (0%)	No: 8/70 (11.4%)	No: 0/58 (0%)

Table 4 reports the results of sub-analysis according to the different hospital setting. The main difference that emerges is that a higher prevalence of dedicated rotation on MSK subspecialties is reported only for University hospitals and larger community hospitals, with *n* = 352/632 (55.7%) and *n* = 74/176 (42%), respectively. Conversely, for private practices and small community hospitals scattered MSK teaching prevailed throughout the various years of residency, with *n* = 36/87 (41.4%) and *n* = 28/66 (42.4%), respectively. As a matter of fact, participants from private practices and small hospitals reported the lowest presence of dedicated rotation period in MSK with *n* = 20/87 (23%) and *n* = 14/66 (21.2%), respectively. Regardless of the hospital setting, more than half of participants rated the quality of MSK training between poor and average, a finding that is in line with that of general survey results. Interestingly, only for private practices the prevalence of answer reporting an “average” quality of MSK training (*n* = 38/86, 44.2%) was superior to “poor” (*n* = 26/86, 30.2%). In the remaining settings, the MSK quality of training was rated mainly as poor, with *n* = 196/632 (31.5%) for university hospitals, *n* = 34.8% (62/178) for larger community hospitals and *n* = 30/66 (45.5%) for small community hospitals. Almost all participants, irrespective of the working setting, believe that the MSK training programme could be improved (percentage > 90% in all settings).

**Table 4:** Table 4 shows the 7 questions with the most heterogeneity of answers, according to the different hospital setting

Question	University hospitals	Larger community hospitals	Private practices	Small community hospitals
6. How is MSK training/teaching organized during your residency programme?	We have a dedicated rotation/block for radiological subspecialties, including MSK: 352/632 (55.7%)	We have a dedicated rotation/block for radiological subspecialties, including MSK: 74/176 (42.0%)	We learn MSK radiology scattered over the various years of residency: 36/87 (41.4%)	We learn MSK radiology scattered over the various years of residency: 28/66 (42.4%)
	We learn MSK radiology scattered over the various years of residency: 167/632 (26.4%)	We learn MSK radiology scattered over the various years of residency: 55/176 (31.0%)	No specialist MSK training is available in our programme: 31/87 (35.6%)	No specialist MSK training is available in our programme: 24/66 (36.4%)
	No specialist MSK training is available in our programme: 113/632 (17.9%)	No specialist MSK training is available in our programme: 47/176 (27.0%)	We have a dedicated rotation/block for radiological subspecialties, including MSK: 20/87 (23%)	We have a dedicated rotation/block for radiological subspecialties, including MSK: 14/66 (21.2%)
9. Do you have/have you had a dedicated period in MSK Ultrasound during your residency?	No: 392/633 (62,0%)	No: 131/175 (74.9%)	No: 65/86 (75.6%)	No: 56/66 (84.8%)
	Yes: 241/633 (38,0%)	Yes: 44/175 (25.1%)	Yes: 21/86 (24.4%)	Yes: 10/66 (15.2%)
10. How many hours of MSK related teaching do you receive annually in your residency programme?	< 20 h: 412/623 (66.1%)	< 20 h: 128/175 (73.1%)	< 20 h 47/85 (55.3%)	< 20 h: 44/64 (68.8%)
	20–40 h: 128/623 (20.5%)	20–40 h: 29/175 (16.6%)	20–40 h: 31/85 (36.5%)	20–40 h: 10/64 (15.6%)
	> 40 h: 83/623 (13.4%)	> 40 h: 18/175 (10.3%)	> 40 h: 7/85 (8.2%)	> 40 h: 10/64 (15.6%)
12. How would you rate your overall MSK training?	Poor: 196/632 (31.5%)	Poor: 62/178 (34.8%)	Average: 38/86 (44.2%)	Poor: 30/66 (45.5%)
	Average: 170/632 (27.3%)	Average: 53/178 (29.8%)	Poor: 26/86 (30.2%)	Average: 20/66 (30.3%)
	Good: 119/632 (19.1%)	Good: 35/178 (19.7%)	Very good: 10/86 (11.6%)	Good: 12/66 (18.2%)
	Very good: 118/632 (18.9%)	Very good: 20/178 (11.2%)	Good: 7/86 (8.1%)	Very good: 3/66 (4.5%)
	Excellent: 29/632 (4.7%)	Excellent: 8/178 (4.5%)	Excellent: 5/86 (5.8%)	Excellent: 1/66 (1.5%)
14. During your training period are/were you expected to learn interventional MSK procedures?	No: 365/631 (57.9%)	No: 117/175 (66.9%)	No: 68/85 (79.1%)	No: 44/66 (66.7%)
	Yes: 266/631 (42.1%)	Yes: 58/175 (33.1%)	Yes: 17/85 (19.8%)	Yes: 22/66 (33.3%)
15. How do/did you participate in interventional MSK procedures during your residency?	As Observer: 230/615 (37.4%)	No MSK procedures are performed in my Institution: 90/176 (51.1%)	No MSK procedures are performed in my Institution: 54/86 (62.8%)	As Observer: 29/66 (43.9%)
	Actively involved: 209/615 (34,0%)	As Observer: 51/176 (29.0%)	As Observer: 21/86 (24.4%)	No MSK procedures are performed in my Institution: 28/66 (42.4%)
	No MSK procedures are performed in my Institution: 176/615 (28.6%)	Actively involved: 35/176 (19.9%)	Actively involved: 11/86 (12.8%)	Actively involved: 9/66 (13.6%)
16. Do you think that MSK training should/could be improved in your residency programme?	Yes: 582/629 (92.5%)	Yes: 161/176 (91.5%)	Yes: 84/86 (97.7%)	Yes: 66/66 (100%)
	No: 47/629 (7.5%)	No: 15/176 (8.5%)	No: 2/86 (2.3%)	No: 0/66 (0%)

## Discussion

The most important finding that emerges from this survey is that more than two-thirds of participants rated the quality of their MSK between “poor” and “average”, with limited time available for dedicated MSK training during the year. It has to be noted that survey results demonstrate considerable heterogeneity through all survey subsets, specifically regarding the structure of MSK training programs, which was evident even within single Countries. Nevertheless, as the survey includes a huge number of answers from young radiologists and trainees from several Countries, it represents an interesting snapshot of the current situation of MSK radiology training across European and Non-European Countries.

Response rates from residents were slightly higher than those of board-certified radiologists. The majority of young board-certified radiologists choose to work in academic institutions or large community hospitals (61%), which probably are better geared to accommodate research, academia and access to more advanced technology. This yet again varies from one country to the other depending on the set up of the national health care systems (see question #5).

Another important aspect that emerges is that relatively little time is dedicated to MSK training within most radiology training programmes. Dedicated MSK training rotations were included in just half of the residency programs (see question #6), and almost 20% of participants reported the absence of any MSK training program. Even when dedicated MSK rotations existed, those were allocated for less than six months in more than two-thirds of responders (see question #7). This paucity of education emerges also from the number of hours related to MSK teaching, which accounts for less than 20 h per year in more than half of participants (see question #10). Adequate MSK training is of paramount importance to general radiologists and those who choose to specialise in MSK, as showed by several studies reporting higher diagnostic accuracy of MSK-trained radiologists. Most plain radiographs (other than chest x-rays) performed for orthopaedic-related disorders, and more than 70% of MRI scans are for spinal or peripheral MSK problems [11]. Focused knowledge is important as MSK subspecialty second-opinion consultation has been proven to be more accurate than generalists reports in 82.0% of examinations; discrepancies were mainly observed in tumour cases [3]. Rozenberg et al. reported significantly higher performance of MSK radiologists when compared with non-MSK radiologists in interpreting orthopaedic oncology examinations, emphasising the importance of subspecialty training [12]. The importance of dedicated MSK training for residents has been recently presented by Nelson et al. by showing significant impact on their ability to report bone densitometry scans and initiate osteoporosis medications [13].

Senior consultants and fellows play a chief role in MSK training (question #11). This may relate to trainees’ preference to learn MSK radiology through daily clinical practice, supervised by experienced radiologists (question #13), a modality that was preferred over formal lecture-based teaching lesson by university professors.

According to the responses received for questions #8 and #9, trainees mostly rotated between different MSK modalities (71% of participants) instead of being assigned to a specific one, with only one-third of them having received dedicated MSK ultrasound sessions. With regards to interventional MSK training, 62% of participants were not expected to learn interventional MSK procedures (question #14); in two-thirds, the procedures were either not performed in their institution or the trainees were asked to be solely involved as observers (question #15). Indeed, the overall quality of MSK training was considered poor-to-average by more than half of participants (question #12), and the vast majority of young radiologists believe that MSK training should be enhanced (question #16), requesting improvements in ultrasound practice, image-guided interventional procedures and MRI interpretation and case discussion (question #17). The need for particular attention to ultrasound is corroborated by the fact that this examination is currently performed by several other specialities, who receive dedicated MSK US training [14]. Worrisome results have shown that that radiology residents in the USA receive far less training in MSK ultrasound than trainees of physical medicine and rehabilitation, sports medicine, and rheumatology [15]. Recent surveys have shown that ultrasound-guided MSK procedures are widely performed across Europe by various practitioners and that ultrasound is preferred as a guidance method over fluoroscopy and CT for joint injections [7, 16, 17]. In this regard, it should be highlighted that the ESSR undertook active measures to improve MSK ultrasound by organising several dedicated courses and publishing new guidelines to standardise clinical practice [18–21]. Both the ESR and ESSR have put efforts to improve the quality of MSK radiology training towards harmonising education across different Countries. In fact, several initiatives have been promoted, such as the European Diploma in Musculoskeletal Radiology (EDiMSK), which is an established qualification aimed at endorsing the skillset of MSK-trained radiologists [6]. Additionally, the European Training Curriculum for Radiology is a continuously updated template aimed to guide trainees in developing basic and in-depth knowledge required for subspecialist training [5]. Unfortunately, too many young ESR radiologists/residents are still not aware of these tools (questions #18 to #20), but when brought to their attention, most of them were highly interested and believe this may improve their recruitment chances in a competitive profession [22]. Our results are in line with those of a very recent survey by the ESSR, which reported the under-recognition of the EDiMSK as it is currently accepted as an official postgraduate qualification in 47% of European Countries [23].

Upon comparing the responses from the top five most represented Countries, we noted some relevant differences. First, while Italy, India, and Portugal had a dedicated rotation in MSK training in 41–50% of cases (concurring with the average in the survey), Spain and UK had dedicated rotations in 100% and 80% of cases, respectively. Spain and UK also had the highest rates of dedicated MSK ultrasound training, education on interventional procedures, hours per year of MSK-related teaching, and overall MSK training opportunities. Nevertheless, almost all participants believed that their MSK training should and could be improved in their residency programme, regardless of the country of residency. This confirms a trend already reported in a recent survey about musculoskeletal radiology training in the UK [24].

The comparison between different hospital settings showed few differences in the relative prevalence of answers, with few exceptions. The most relevant is probably the discrepancy in the prevalence of dedicated rotation on MSK subspecialties, which was higher for university and larger community hospitals. A possible explanation of this may be related to the more structured residency programs in the university departments, or the subdivision into more specialised branches often seen in larger hospitals even within radiology departments.

This survey has some limitations. Firstly, this was not an all-encompassing survey, since we could not consider a number of factors related to different health care systems and University programs (e.g., years of residency) of the different Countries that may have in turn affected the results [17]. Second, the methodology of this study based on a questionnaire introduces bias to the results through subjective evaluation of the problem. Nevertheless, in order to limit bias as much as possible we included only 2 questions (#12 and #16) that allowed a “subjective” answer. All the remaining questions were related to the objective structure of MSK training and therefore, cannot be influenced too much by the type of training experienced. In addition, the survey was sent to individual residents and radiologists and not to residency programs, thus may limit information about how many and which programs are represented. Our study design aimed to avoid stakeholders and conflict of interest bias for example if program directors or any of the decision makers would have been asked the same questions, this would have almost certainly influenced the results in the direction of overcompensating and not registering issues. Thus, it is essential to interview the person who is most involved in the outcome of the training as a stakeholder for personal future endeavours and everyday clinical work. Nevertheless, we chose to approach and collect individual experiences, to ensure anonymity of participants and to obtain unbiased results. Finally, the survey did not include questions about the level of residency training/number of training years, an information that may have further put the overall results into perspective by reflection; however, this would not have influenced the objective data collected. There are limitations inherent to the study design as we set out the ambitious task evaluating quite a complex scenario such as the global level of MSK training among young radiologists.

In conclusion, there are significant inconsistencies in the structure of MSK training offered by different Countries. Nonetheless, there is a unified need to improve MSK training in all residency programs, as advocated by the great majority of participants who demand a special attention to MSK ultrasound, MRI reporting, and image-guided interventional procedures. In recognition of MSK as a radiological subspecialty which is increasingly popular, both the ESR and ESSR have led initiatives to standardise and enhance MSK radiology training, promote standards of excellence and attract future generations to join our profession. The ESR and ESSR play a pivotal role in leading this strategic goal by opening continuous channels for education, quality assurance and support for radiologists who choose to train and work in MSK radiology.

## Data Availability

The datasets used and/or analysed during the current study are available from the corresponding author on reasonable request.

## Abbreviations

EDiMSK: European Diploma in Musculoskeletal Radiology
ESR: European Society of Radiology
ESSR: European Society of Musculoskeletal Radiology
MSK: Musculoskeletal
RTF: Radiology Trainees Forum
